# Feedback Modulates Audio-Visual Spatial Recalibration

**DOI:** 10.3389/fnint.2019.00074

**Published:** 2020-01-17

**Authors:** Alexander Kramer, Brigitte Röder, Patrick Bruns

**Affiliations:** Biological Psychology and Neuropsychology, University of Hamburg, Hamburg, Germany

**Keywords:** crossmodal learning, crossmodal recalibration, sound localization, ventriloquism aftereffect, supervised learning, multisensory, feedback, spatial perception

## Abstract

In an ever-changing environment, crossmodal recalibration is crucial to maintain precise and coherent spatial estimates across different sensory modalities. Accordingly, it has been found that perceived auditory space is recalibrated toward vision after consistent exposure to spatially misaligned audio-visual stimuli (VS). While this so-called ventriloquism aftereffect (VAE) yields internal consistency between vision and audition, it does not necessarily lead to consistency between the perceptual representation of space and the actual environment. For this purpose, feedback about the true state of the external world might be necessary. Here, we tested whether the size of the VAE is modulated by external feedback and reward. During adaptation audio-VS with a fixed spatial discrepancy were presented. Participants had to localize the sound and received feedback about the magnitude of their localization error. In half of the sessions the feedback was based on the position of the VS and in the other half it was based on the position of the auditory stimulus. An additional monetary reward was given if the localization error fell below a certain threshold that was based on participants’ performance in the pretest. As expected, when error feedback was based on the position of the VS, auditory localization during adaptation trials shifted toward the position of the VS. Conversely, feedback based on the position of the auditory stimuli reduced the visual influence on auditory localization (i.e., the ventriloquism effect) and improved sound localization accuracy. After adaptation with error feedback based on the VS position, a typical auditory VAE (but no visual aftereffect) was observed in subsequent unimodal localization tests. By contrast, when feedback was based on the position of the auditory stimuli during adaptation, no auditory VAE was observed in subsequent unimodal auditory trials. Importantly, in this situation no visual aftereffect was found either. As feedback did not change the physical attributes of the audio-visual stimulation during adaptation, the present findings suggest that crossmodal recalibration is subject to top–down influences. Such top–down influences might help prevent miscalibration of audition toward conflicting visual stimulation in situations in which external feedback indicates that visual information is inaccurate.

## Introduction

When spatially interacting with our environment, vision and audition communicate in multifaceted ways to guide attention (Driver and Spence, 1998), enhance spatial acuity (Bolognini et al., 2007), and form a coherent representation of our environment. In order to benefit from multiple sensory sources, the signals must be integrated across sensors. Spatial proximity is one of the main cues to decide whether or not two signals belonged to the same event (Holmes and Spence, 2005). In the case of audio-visual spatial perception, assessing spatial proximity is a strikingly complex task, as spatial representations in vision are directly provided by the retina (in eye-centered coordinates), whereas in audition spatial cues emerge from the interaction of the sound waves with the head (Mendonça, 2014) and have to be transformed into a (head-centered) spatial code. It has been argued that the perceptual system uses vision to calibrate auditory spatial perception due to its usually superior spatial resolution and, thereby, resolves misalignments between sensory representations (Radeau and Bertelson, 1974; Knudsen and Knudsen, 1989; Bertelson et al., 2006; King, 2009; Kopco et al., 2009). Misalignments between sensory representation typically arise during development due to changes in interocular and interaural distance and head size. However, multisensory calibration is not limited to development but rather a lifelong process (Gilbert et al., 2001).

A vivid example of crossmodal recalibration in adults is the ventriloquism aftereffect (VAE), in which exposure to audio-visual stimuli (VS) with a consistent spatial discrepancy induces a subsequent shift in unisensory auditory localization (Radeau and Bertelson, 1974). The VAE can be induced with various audio-visual exposure durations ranging from a single exposure (Wozny and Shams, 2011; Bruns and Röder, 2015) over an exposure lasting for several minutes (Recanzone, 1998; Lewald, 2002; Bruns et al., 2011) to several days (Zwiers et al., 2003). With longer adaptation times, the size of the aftereffect increases (Frissen et al., 2012). The size of the aftereffect is usually only a fraction of the original audio-visual discrepancy (10–50%) (Bertelson et al., 2006; Kopco et al., 2009; Frissen et al., 2012). More drastic interventions such as the use of prisms over days (Zwiers et al., 2003) to weeks (Bergan et al., 2005) while continuously interacting with the environment have been shown to result in a stronger and more complete realignment of audition with the new visual world.

In case of the VAE, the mere existence of an audio-visual discrepancy implies that at least one of the sensory estimates must be inaccurate. However, without external feedback, the perceptual system cannot infer which sensory estimate was inaccurate and, thus, which sensory representation should be recalibrated (Zaidel et al., 2013). While the VAE as a form of recalibration manifests in subsequent unisensory shifts, auditory localization is also biased toward vision during audio-visual stimulation, referred to as the ventriloquism effect (VE). Studies investigating such immediate effects as examples of multisensory integration have found that a unified multisensory percept is formed as a weighted average based on the precision of the individual cues, which is considered optimal since such a combination rule maximizes the precision of the multisensory percept (Ernst and Banks, 2002; Alais and Burr, 2004). It has been demonstrated that auditory localization accuracy is positively correlated with precision along the horizontal plane (Garcia et al., 2017). If accuracy is correlated with precision and precision is directly accessible to the perceptual system (Ernst and Di Luca, 2011), some authors have argued that it would be optimal if recalibration was based on the reliability of the individual cues, too (reliability-based adaptation, for examples see Ghahramani et al., 1997; van Beers et al., 2002; Burge et al., 2010; Makin et al., 2013). However, precision does not necessarily imply accuracy (Ernst and Di Luca, 2011). Thus, several authors have argued that the perceptual system forms prior beliefs about the accuracy of individual senses which are independent of precision (Block and Bastian, 2011; Ernst and Di Luca, 2011). Recalibration is then assumed to be based on the prior beliefs about accuracy rather than on current reliability. Accordingly, it has been proposed that sensory estimates are adapted according to a fixed ratio (fixed-ratio adaptation) which is relatively stable over time and independent of short-term variations in sensory precision (Zaidel et al., 2013). Crossmodal recalibration consistent with a fixed-ratio adaptation was indeed observed in visual-vestibular motion perception (Zaidel et al., 2011).

Regardless of whether recalibration is reliability-based or follows a fixed-ratio, it would lack external validation in a purely sensory context in which accuracy can only be inferred either from the same cues that are subject to recalibration, which would be circular, or from prior beliefs that can turn out to be wrong when the environment changes. Several authors have argued that this circularity can only be overcome by the use of external feedback which provides independent information about the state of the world (Di Luca et al., 2009; Zaidel et al., 2013). While it is known that unisensory and sensorimotor perceptual learning is susceptible to external feedback (Adams et al., 2010), to our knowledge only one study has investigated whether crossmodal recalibration is modulated by external feedback (Zaidel et al., 2013).

Zaidel et al. (2013) demonstrated that, unlike recalibration without external feedback (unsupervised recalibration), crossmodal recalibration depended on cue reliability when external feedback about the sensory accuracy was provided which was based on the spatial location of one of the two sensory cues (supervised recalibration). In a visual-vestibular motion VAE paradigm, Zaidel et al. (2013) manipulated visual reliability such that it was either set higher or lower than vestibular reliability. Feedback was either given based on motion implied by visual motion stimuli or based on vestibular motion stimuli which were presented simultaneously. Whereas unsupervised recalibration was independent of cue reliability (Zaidel et al., 2011), supervised recalibration was found to be based on the discrepancy between the multisensory (i.e., integrated) percept and the location indicated by feedback. As the multisensory percept in visual-vestibular motion perception is highly dependent on cue reliability (Gu et al., 2008; Fetsch et al., 2009) supervised recalibration therefore also depended on cue reliability. Zaidel et al. (2013) argued that both mechanisms together result in accurate, precise and consistent multisensory and unisensory representations of space. The idea is that unsupervised recalibration aligns sensory modalities, thereby providing a consistent representation of space, and supervised learning realigns this internally consistent representation with the external world.

However, in order to accept these ideas as a general rule, it has to be demonstrated that they hold for other combinations of sensory modalities such as for audio-visual stimulation. In fact, empirical results have suggested that audio-visual spatial recalibration in the VAE might be unaffected by top–down processes. For example, the VAE did not differ between audio-visual trials which included matching voices and faces or percussion sounds and a video of hands playing bongo, compared to trials in which the VS was simply a synchronously modulated diffuse light (Radeau and Bertelson, 1977, 1978). Furthermore, although attentional load was found to influence the spatial pattern of the VAE, the overall size of the VAE remained unaffected (Eramudugolla et al., 2011). These results were taken as evidence for the idea that the VAE is largely independent of top–down effects such as attention. In accordance with this proposal are findings that the VAE occurs even when participants are asked to ignore VS or become aware of the audio-visual discrepancy (Bertelson, 1999). However, it is not known whether the VAE is modulated by external feedback regarding the spatial accuracy of either the auditory or visual cue. In fact, such feedback would be a crucial prerequisite to guarantee external accuracy of perception, that is, a correct relation between sensory representations and the external world.

In order to test whether crossmodal recalibration is affected by external spatial feedback, we extended the classical VAE paradigm (Radeau and Bertelson, 1974; Recanzone, 1998) by introducing feedback similar to that employed by Zaidel et al. (2013). During an audio-visual block, participants had to localize audio-VS with a fixed spatial discrepancy. In contrast to previous studies, feedback about the localization error was provided. Each participant completed four sessions and in half of the sessions feedback in audio-visual blocks was calculated based on the discrepancy between the participant’s response and the true visual position, and in the other half of the sessions feedback was based on the discrepancy between the participant’s response and the true auditory position.

As there are a few reports of visual aftereffects in the ventriloquism paradigm (Radeau and Bertelson, 1976; Lewald, 2002) which could potentially be increased by feedback that is based on the auditory stimulus (AS) position, we tested both auditory and visual unimodal localization before and after the audio-visual block to assess both auditory and visual aftereffects. Based on the assumption that feedback would update the perceptual system’s beliefs about the accuracy of the involved sensory cues, we hypothesized that the VAE would decrease for the sensory modality that feedback was based on. The opposite effect was expected for the other modality for which feedback did not indicate the true stimulus location. Moreover, as accuracy was found to be correlated with precision in audition (Garcia et al., 2017) and precision modulated effects of feedback in visual-vestibular recalibration (Zaidel et al., 2013), we additionally tried to manipulate the reliability of the VS. In accordance with Zaidel et al. (2013), we hypothesized that recalibration in the presence of feedback is based on relative cue reliabilities. Hence, the VAE would be increased for the less reliable sensory modality.

## Materials and Methods

### Participants

In order to counterbalance all control conditions (see section “Procedure” for details), we were restricted to multiples of 24 for our sample size. We aimed for a sample size of 24 participants, which has 80% power (at an α level of 0.05) to detect a medium-sized effect (*d*_*z*_ = 0.52) for a directional difference between two within-subject conditions (corresponding to our main hypothesis that the VAE is reduced when feedback is based on the auditory position rather than on the visual position). The power analysis was conducted in G^∗^Power 3.1 (Faul et al., 2009).

A total of 37 healthy adult volunteers were recruited through an online subject pool of the University of Hamburg, because 13 datasets had to be removed from the initial sample due to technical issues which led to a wrong presentation of AS locations. All affected datasets were replaced such that complete datasets from 24 participants were acquired. At the analysis stage, six additional datasets had to be excluded from the 24 participants which completed all sessions. One participant reported visual field restrictions in one hemifield after completion of the experiment and had to be removed from the sample. Moreover, five participants had to be removed due to untypically inaccurate responses or poor performance in catch trials (see section “Data Analysis” for details).

The remaining 18 participants (4 males, 14 females) were from 19 to 39 years of age (mean: 24.4 years) and reported normal hearing and normal or corrected-to-normal vision. Participants received course credits as compensation. Additionally, participants received monetary rewards (mean = 25.56€, possible minimum = 0€, possible maximum = 46.80€, empirical minimum = 17.55€, empirical maximum = 39.60€) as part of the experiment. Written informed consent was obtained from all participants prior to taking part. The study was performed in accordance with the ethical standards laid down in the 2013 Declaration of Helsinki. The procedure was approved by the ethics commission of the Faculty of Psychology and Human Movement of the University of Hamburg.

### Apparatus

**FIGURE 1:**
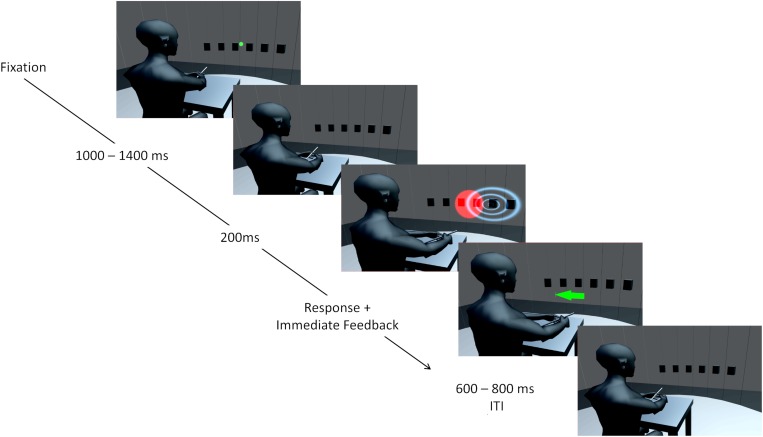
Illustration of the setup and an audio-visual trial. Six speaker positions from –22.5 to 22.5° in steps of 9° are represented by black boxes. The curtain covering the speakers is only transparent for illustration purposes and was visually opaque and only acoustically transparent. A chin rest used to fixate the head is not displayed. At first, a green laser dot appeared as fixation point and participants could start the trial by pointing to the fixation dot and pressing a button. The trial started when the pointing error was below ± 10°. During a second interval, a step motor adjusted a second laser used for stimulus presentation. Auditory (indicated by blue waves) and visual (red light cone) stimuli were presented for 200 ms in synchrony. Participants could respond immediately by pointing toward the perceived direction and pressing a button on the pointer. Corrective feedback followed instantaneously in form of a centrally presented arrow. The color of the arrow (green for reward, red for no reward) and a unique sound indicated whether a reward was obtained. After a varying interval (600–800 ms) the green laser dot reappeared, and the participant could start the next trial. Avatar image adapted from “Low Poly Character” by TehJoran (2011) (https://www.blendswap.com/blend/3408) licensed under CC BY.

Experiments were conducted in a sound-attenuated and darkened room. Participants were seated in the center of a semicircular frame (90 cm radius) on which six loudspeakers were mounted at ear level. Hence, all auditory stimuli were presented at the same height. Loudspeaker locations ranged horizontally from 22.5° left from straight-ahead (0°) to 22.5° right from straight-ahead in steps of 9° (−22.5, −13.5, −4.5, 4.5, 13.5, and 22.5°). Participants positioned their head on a chin rest to fix the head position across trials. An acoustically transparent curtain covered the loudspeakers. A schematic illustration of the apparatus is shown in Figure 1. Visual stimulation was provided via four laser pointers which projected a light point onto the curtain for 200 ms. Two laser beams were diffused resulting in circular red light blobs with approximately Gaussian luminance amplitude envelopes. The sizes (horizontal and vertical) of the VS, defined by the standard deviation of the luminance distribution, were 12.84° for the low reliable VS and 2.83° for the high reliable VS. The position of a VS was defined as the center of its luminance distribution. The center of the luminance distribution in the vertical dimension was always at the same height as the speakers. A third and fourth laser pointer were not diffused and purple and green in color. The laser pointers were mounted on a step motor with an angular resolution of 0.9° and a horizontal range of 180°. Auditory stimuli were narrow-band filtered (1/2 octave) pink noise bursts with four different center frequencies (250, 500, 1000, or 2000 Hz) and were presented for 200 ms including 5 ms on- and off-ramps. The stimulus intensity was randomly varied over a 4-dB range centered at 70 dB(A) to minimize potential differences in the loudspeaker transformation functions. Participants localized stimuli with a custom-build pointing stick which recorded azimuthal position with 1° resolution.

To deliver feedback, an LED-panel (APA 102, Shiji Lighting, Shenzhen, China) measuring 32 cm in width and 8 cm in height with a pixel width of 0.5 cm and a spacing of 0.5 cm (2.54 ppi) was attached to the semi-circular frame between ± 10.2° azimuth and 2 cm below the lower edge of the loudspeakers. An Arduino Leonardo (Arduino SRL, Strambino, Italy) was used to interface between the experimental computer and the LED-panel.

### Procedure

**FIGURE 2:**
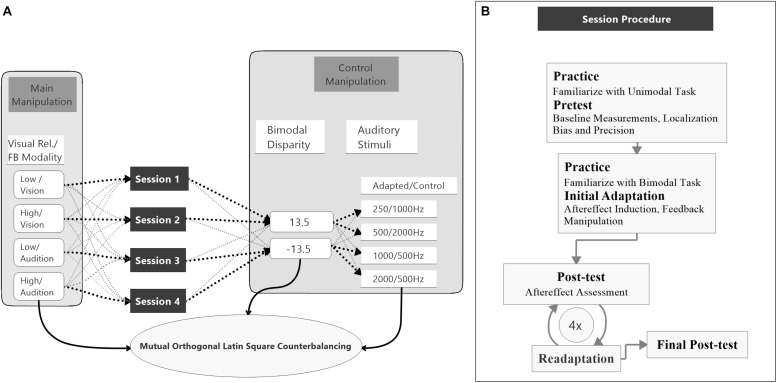
Study design and session procedure. **(A)** The flow diagram shows the counterbalancing procedure. An exemplary procedure for one participant is depicted with bold black pointed lines. All possible assignments between the main conditions, session number, bimodal disparity, and auditory stimulus (AS) pair are depicted with light gray pointed lines. Assignments of main conditions to session number, bimodal disparity and AS pairs were mutually counterbalanced by orthogonal Latin squares. **(B)** The flow diagram visualizes the procedure of a single session. All four sessions were performed following the same procedure.

The study was split into four sessions which were conducted on separate (but not necessarily consecutive) days (see Figure 2A). Each session started with a unimodal pretest to measure baseline localization accuracy and precision for VS and auditory stimuli presented in isolation. Afterward, an audio-visual adaptation block (see below) was conducted to induce auditory and potentially visual VAEs. The adaptation block was followed by unimodal test blocks to assess the magnitude of the aftereffects. To ensure that aftereffects did not decay over unimodal test blocks, each test block was preceded by a short re-adaptation block. The general procedure of a session is illustrated in Figure 2B.

Two factors were varied between sessions, the reliability of the VS (manipulated by the size of the circular light cone) and the feedback modality. During adaptation blocks participants were asked to localize the AS and feedback about the magnitude and direction of their localization errors was provided. Error feedback was consistently calculated either based on the position (i.e., center of the luminance distribution) of the VS (vision feedback modality) or based on the position of the auditory stimuli (audition feedback modality) within each session. All participants completed all combinations of visual reliability (high vs. low) and feedback modality (vision vs. audition) across sessions. The auditory stimuli were grouped into four pairs (250 Hz/1000 Hz, 500 Hz/2000 Hz, 1000 Hz/250 Hz, 2000 Hz/500 Hz) with non-overlapping frequency spectra. The first stimulus of each pair was the adapted AS and was used during both unimodal blocks and audio-visual adaptation blocks. The second stimulus was only used during the unimodal blocks and served as a control stimulus (CS). Thereby, the CS allowed to test for a sound-frequency transfer of the aftereffect. Each session was conducted with a unique pair of auditory stimuli to avoid carry-over effects between session (Bruns and Röder, 2019a).

Moreover, to avoid that participants became aware of the audio-visual discrepancy during adaption blocks and, thus, might apply explicit response strategies, in half of the sessions the VS were consistently displaced to the left and in the other half to the right of the sound source. To avoid effects of session order, AS assignment or visual discrepancy direction on the feedback modality and reliability conditions, these factors were counterbalanced across participants using a mutual orthogonal Latin square design (Julian et al., 1996). For factors with four levels (discrepancy was dummy coded by taking each discrepancy twice) three mutual orthogonal 4 × 4 Latin squares exist, so that there were six possible ways of assigning Latin squares to the three factors (session order, AS assignment, visual discrepancy direction). As four participants are necessary to realize one Latin square, in total 24 participants were necessary for a balanced design that realizes all combinations of Latin squares. However, factors relevant for the data analysis (visual reliability and feedback modality) were measured within-subject and, thus, were counterbalanced irrespective of participant exclusion (see section “Data Analysis” for details).

### Unimodal Blocks

Unimodal pre- and post-tests were identical, except that the post-test was split into several blocks. The two auditory stimuli (AS, CS) were presented from all six speakers (−22.5, −13.5, −4.5, 4.5, 13.5, and 22.5°). One VS was presented from the same six positions as the auditory stimuli. Either the low reliable VS or the high reliable VS was consistently used across the whole session according to the counterbalancing procedure. The VS was described to participants as a diffuse light cloud and they were instructed to localize the center of this light cloud. For each position and stimulus type (AS, CS, and VS) 10 trials were presented, yielding 180 trials in total. For the pretests, all 180 trials were presented in a random order. For the post-tests, the 180 trials were split into five blocks of 36 trials each. Two trials per position and stimulus type were presented in each block of the post-test. Each trial started with the presentation of a green fixation laser point at 0° azimuth. Participants were required to direct the pointing stick toward the fixation point and started the trial by a button press. The trial only started when the pointing direction deviated less than ± 10 from 0°. This procedure assured a constant starting position for all pointing movements. After a random delay between 400 and 600 ms the presentation of the VS was prepared: the step motor carrying the laser pointer was first moved to a random position between −50 and 50° and then moved to the target position. This was done to avoid that the duration of the sound evoked by the moving step motor provided a cue for the VS position. After another delay of 600 to 800 ms, the VS was presented. During AS and CS trials only a random delay between 1000 and 1400 ms was used after fixation, followed by the presentation of the stimuli. Responses were allowed immediately after stimulus onset. Participants were instructed to respond fast and accurately, but to prioritize accuracy over response speed. Moreover, participants were informed that all stimuli (during unimodal and audio-visual blocks) would be displayed at the same height and that they should focus on localizing stimuli accurately in the horizontal plane. No feedback or reward was provided during pre- and post-test trials. Between trials a random delay between 600 and 800 ms was introduced.

### Audio-Visual Blocks

In order to induce the VAE, the AS and the VS were synchronously presented for 200 ms with a spatial displacement of the VS of either 13.5° to the left or 13.5° to the right of the sound location. The spatial discrepancy was constant during a session. In the initial audio-visual adaptation block, stimuli were presented 20 times at each of six positions (sound at −22.5, −13.5, −4.5, 4.5, 13.5, and 22.5°). The four audio-visual re-adaptation blocks (prior to each of the following unimodal post-test blocks) only contained 10 trials per position and were conducted to counteract a potential decay of the aftereffect (for similar procedures, see Bruns et al., 2011; Zierul et al., 2017). Overall, each session included 360 audio-visual adaptation trials and 360 unimodal test trials (720 trials in total). Participants were instructed to localize the sound (i.e., to ignore the visual location) in audio-visual trials. Immediately after the response, feedback about the azimuthal localization error the was given. The localization error was either calculated as the deviation of the azimuthal pointing direction from the true azimuthal location of the AS or as the deviation of the azimuthal pointing direction from the true azimuthal location of the VS. The modality used for calculating the localization error was held constant within a session. Feedback consisted of a centrally presented arrow with the origin at 0° and heading in the direction participants had to correct their localization response to in order to reduce the error. The length of the arrow equaled the magnitude of the localization error in cm rounded to the next integer, with an upper bound of 16 cm (10.2°) and a lower bound of 4 cm (2.55°). Errors below 4 cm (2.55°) were indicated with a filled circle with a radius of 3 cm (1.9°). Furthermore, participants received a monetary reward (0.03€) when the error fell below an individual threshold which was set to the participant’s 30^th^ percentile of the absolute localization error in the auditory trials of the pretest. A reward was indicated by a unique sound (400 ms custom rebuild of the Super Mario coin sound effect) and a green feedback arrow or circle. A localization error above the individual threshold was indicated by another unique sound (300 ms tone that changed pitch from 100 to 60 Hz after 150 ms) accompanied by a red feedback arrow. The whole sequence of an audio-visual trial is depicted in Figure 1. After each block participants were informed about the amount of reward they had collected during the block. The total amount of reward was disbursed at the end of the session.

In order to assure that participants attended to both visual and auditory stimuli, deviant trials were presented intermixed between regular trials with a probability of 0.1. In deviant trials, participants were instructed to localize a laser point as fast and accurately as possible that differed in color (purple) and was not accompanied by a sound. The laser point was presented until a response was given. When the reaction time fell below the 50^th^ percentile of the reaction time in visual trials of the pretest and localization error was less than 5°, a reward (0.03€) was earned in these trials. The same visual and auditory feedback was used as for regular trials, except that always circular shapes were used.

### Data Analysis

Data were acquired for 24 participants in order to counterbalance control conditions (session order, stimulus assignment, and audio-visual disparity). However, overall six participants had to be excluded from further analyses. One participant reported partial vision in one hemifield after the study was completed. Another two participants failed to respond properly to audio-visual deviant trials. The deviant trials required participants to respond fast and accurately (see section “Audio-Visual Blocks” for details) to receive a reward. Hence, not attending to the VS or closing the eyes during audio-visual blocks would lead to a low amount of rewards in deviant trials. These two participants consistently received rewards in less than 2% of the deviant trials across all sessions, whereas on average participants received rewards in 55% (minimum = 15%, maximum = 82%) of the deviant trials. Hence, we excluded their data from further analyses. For each of the remaining participants we fitted linear models between true azimuthal stimulus positions and azimuthal localization responses for each session and each stimulus (a slope of one and an intercept of zero indicate perfect localization). Three participants with either a slope or an intercept that differed three standard deviations from the mean of all participants were excluded as this indicated an extremely inaccurate localization behavior. All further data analyses were based on the data of the remaining 18 participants.

Importantly, all factors relevant for further data analyses (i.e., Feedback Modality and Visual Reliability) were still fully counterbalanced after exclusion of the participants. The reduction of the sample size only affected the counterbalancing of session order, assignment of sound pairs to sessions and assignment of audio-visual discrepancy directions to sessions. The final numbers of participants for each combination of these factors are summarized in Supplementary Tables 1–3.

To test whether participants changed their localization behavior in audio-visual adaptation trials according to the error feedback, we took the mean localization error in the first 10 adaptation trials of the initial adaptation block and compared this score with the mean localization error of the last 10 adaptation trials in the last re-adaptation block. We performed two separate *t*-tests for the conditions of feedback modality (audition or vision) comparing the mean of the first 10 trials to the mean of the last 10 trials.

Measurements for accuracy and reliability were derived from unimodal blocks and based on a common model of measurement error (Grubbs, 1973). Each trial is interpreted as a measurement *y*_*ik*_ for the true stimulus position *x*_*k*_ where *i* is an index over the trial numbers and *k* over stimulus positions. The measurement model is then formalized as

(1) $$y_{\mathrm{ik}}=x_{k}+a_{k}+e_{\mathrm{ik}},$$

were *a*_*k*_ is a constant bias for the *k*th stimulus position and *e*_*ik*_ are independent mean zero random errors. As an estimator for accuracy we calculated the constant error ${\hat{a}}_{k}$ by averaging localization responses of all trials for each combination of stimulus position, condition and participant. For a given stimulus position this is a robust estimator of the bias term *a*_*k*_ and thus accuracy. We will further refer to $\hat{a}:=M\,\left({\hat{a}}_{k}\right)$ as *constant bias*, which is an overall measure for the tendency to systematically mislocalize in one direction across all locations. Reliability is defined as the inverse of the variance of *e*_*ik*_. Due to the direct relation between variance and reliability we assessed the *variable error*, a robust estimator of the variance (Levene, 1960), as a measure for reliability. The variable error is defined as the mean absolute deviation of the localization response from the mean localization response for a given stimulus position, that is, if $\hat{y_{i\,k}}$ are the participant’s responses the variable error is defined as $M\,\left(\left|\hat{y_{i\,k}}-\,{\hat{a}}_{k}\right|\right)$. A high variable error indicates a low reliability and vice versa.

First, we tested whether we were successful in manipulating the reliability of the VS (high or low) and controlled that auditory reliabilities did not differ prior to adaptation. Therefore, variable errors calculated from all pretest trials were submitted to a repeated measures MANOVA (O’Brien and Kaiser, 1985) with factors Feedback Modality (audition or vision), Stimulus Type (AS, CS, and VS), Stimulus Position (−22.5, −13.5, −4.5, 4.5, 13.5, and 22.5°) and Visual Reliability (low or high). This approach is not affected by violations of the sphericity assumption and allows for *post hoc* interaction contrasts, which were conducted to further analyze significant MANOVA effects.

The VAE was measured as change in the constant bias between pre- and post-test blocks. For this purpose, data from the five post-test blocks were pooled. More specifically, the difference of post-test constant bias (${\hat{a}}_{p\,o\,s\,t}$) and pretest constant bias (${\hat{a}}_{p\,r\,e}$) multiplied with the sign of the audio-visual discrepancy (Diff_*AV*_) was taken as a measure for the VAE, thus VAE = ${\left({\hat{a}}_{p\,o\,s\,t}-{\hat{a}}_{p\,r\,e}\right)}^{*}\,s\,i\,g\,n\,\left({\text{Diff}}_{A\,V}\right)$ (for a similar procedure see Bruns and Röder, 2019b). This procedure assured that aftereffects in the direction of the VS always had a positive sign irrespective of whether the VS was displaced to the left (−13.5°) or to the right (13.5°). The resulting values were submitted to a repeated measures MANOVA (O’Brien and Kaiser, 1985) with Feedback Modality (audition or vision), Stimulus Position (−22.5, −13.5, −4.5, 4.5, 13.5, and 22.5°) and Stimulus Type (AS, CS, and VS) as within-subject factors.

## Results

### Unimodal Precision

**FIGURE 3:**
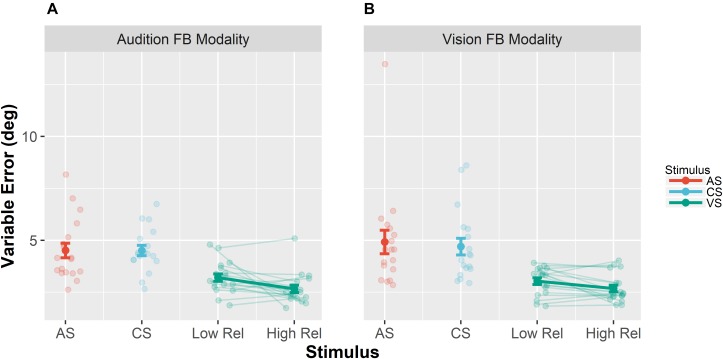
Mean variable errors in the pretest. Variable errors were defined as absolute trial-wise deviation from the mean localization response, averaged across stimulus positions and participants. **(A)** Results when audition was the feedback modality. **(B)** Results for vision as the feedback modality. Each panel shows the variable error separately for the different stimuli [adapted sound (AS), control sound (CS), and visual stimulus (VS)]. Moreover, results for the VS are shown separately for the VS with low reliability (Low Rel) and high reliability (High Rel). Individual data are shown with light-colored points and lines, whereas sample averages are indicated by dark-colored points and bold lines. Paired data points (i.e., individual data from a single participant) are connected via lines. Error bars represent standard error of the mean. Mean values are depicted on top of each bar.

Unimodal pretests were performed in order to assess localization biases and reliabilities for all stimulus types and positions. We evaluated whether we succeeded in manipulating the visual reliability and whether auditory reliability significantly differed across conditions at baseline. Therefore, variable errors at pretest (see section “Data Analysis” for a definition) were submitted to a repeated measures MANOVA (O’Brien and Kaiser, 1985) with factors Feedback Modality (audition or vision), Stimulus Type (AS, CS, and VS), Stimulus Position (−22.5, −13.5, −4.5, 4.5, 13.5, and 22.5°) and Visual Reliability (low vs. high). Only a main effect of Stimulus Type was found, *F*(1,17) = 35.22, *p* < 0.001, showing that visual reliability was higher than auditory reliability independent of the reliability manipulation (see Figure 3). Since no main effect of visual reliability was found (see Table 1 for full results), this factor was not further considered in the following analyses.

**TABLE 1:** Repeated measures MANOVA on variable errors in the pretest.

**Effect**	**Num Df**	**Den Df**	**Pillai test statistic**	**Approximately *F***	***p***
Intercept	1	17	0.93	249.66	<0.001
Feedback modality	1	17	0.04	0.11	0.43
Visual reliability	1	17	0.01	0.03	0.74
Stimulus type	1	16	0.81	27.49	<0.001
Feedback modality: visual reliability	1	17	0.06	0.54	0.29
Feedback modality: stimulus type	1	16	0.10	1.47	0.43
Reliability: stimulus type	1	16	0.21	1.92	0.14
Feedback modality: reliability: stimulus type	1	16	0.04	0.39	0.70

Additionally, we performed pairwise contrasts to assess whether the variable error changed from pre- to post-test separately for all stimulus types (AS, CS, and VS). Results are summarized in Table 2. Importantly, the variable error did not decrease for auditory stimuli (AS and CS), but it decreased for the VS, both when audition was the feedback modality, *F*(1,17) = 16.75, *p* < 0.001, and when vision was the feedback modality, *F*(1,17) = 6.43, *p* = 0.021.

**TABLE 2:** Pairwise contrasts for auditory variable errors between pre- and post-test.

**Contrast**	**Stimulus**	**FB-modality**	**Mean variable error at pretest**	**Mean difference**	**Pillai test statistic**	**Approximately *F***	**Num Df**	**Den Df**	***p***
Post - pre	AS	Audition	4.51	–0.14	0.011	0.20	1	17	0.663
Post - pre	AS	Vision	4.92	–0.02	<0.001	<0.01	1	17	0.942
Post - pre	CS	Audition	4.51	0.16	0.028	0.51	1	17	0.487
Post - pre	CS	Vision	4.70	0.47	0.20	4.33	1	17	0.053
Post - pre	VS	Audition	2.93	–0.49	0.50	16.75	1	17	<0.001
Post–pre	VS	Vision	2.86	–0.25	0.27	6.43	1	17	0.021

*All *p*-values are uncorrected.*

Moreover, a contrast was performed to test whether in the post-test blocks the variable error differed for the AS between the conditions audition feedback modality (*M* = 4.4°, *SD* = 1.3°) and vision feedback modality (*M* = 4.9°, *SD* = 1.9°). No significant difference was found, *F*(1,17) = 2.50, *p* = 0.132.

### Audio-Visual Blocks

**FIGURE 4:**
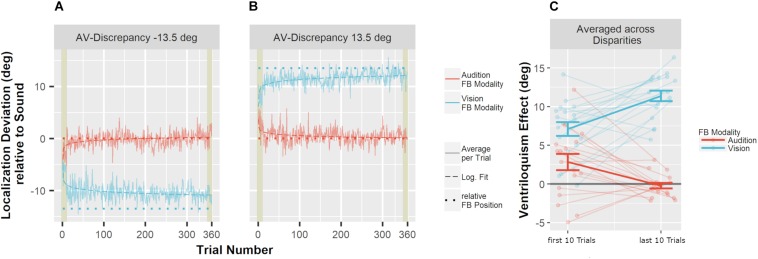
Mean localization deviations in audio-visual adaptation blocks. **(A,B)** Averages across participants and stimulus positions for each adaptation trial are displayed depending on whether audition (red) or vision (blue) was the feedback modality. Mean deviations were derived by averaging across all participants for one specific trial. The trial number reflects the order of the trials during audio-visual blocks. The position of the sound was used as reference (relative position of 0°). Sessions including an audio-visual discrepancy to the left (–13.5°) are depicted in **(A)**, and sessions with a discrepancy to the right (13.5°) are depicted in **(B)**. The actual data (solid line) were logarithmically interpolated (dashed line) to visualize the trend across trials. The relative position that was used to calculate error feedback is indicated by the dotted lines (rel. FB Position). In all conditions, participants adjusted their localization behavior in the direction implied by the error feedback. Participants started with an offset toward the visual position which reflects the well-known ventriloquism effect. The first and last 10 trials are highlighted by khaki rectangles. These trials were averaged per participant for statistical analyses. **(C)** Localization deviations averaged across the first 10 and the last 10 audio-visual adaptation trials. Individual data are shown with light-colored points and lines whereas sample averages are indicated by dark-colored bold lines. Paired data points (i.e., individual data from a single participant) are connected via lines. Error bars represent the standard error of the mean. The effect of feedback was very prominent already within the first 10 trials **(A,B)**. As a consequence, localization responses already differed at baseline (i.e., over the first 10 trials) depending on whether audition or vision was the FB modality **(C)**. Nevertheless, a comparison of the first 10 trials and the last 10 trials demonstrated a clear effect of FB modality (see text for details).

To test whether feedback altered auditory localization in bimodal trials during adaptation, we calculated the difference of the auditory localization response from the true auditory position. The VE was apparent in a shift of auditory localization toward the accompanying VS (Figure 4). Crucially, when feedback was given based on to the true auditory position, the VE decreased over the course of the adaptation trials. In contrast, feedback based on the visual position increased the VE. To statistically test the change of the VE size over the course of the audio-visual adaptation trials, we calculated the means of the first 10 trials and the means of the last 10 trials in the audio-visual blocks, multiplied with the sign of the audio-visual discrepancy (thus, a shift of auditory localization toward the VS was always positive). These values were compared with Bonferroni–Holm corrected paired-sample *t*-tests. Feedback based on to the auditory position significantly decreased the VE from the first 10 trials of the audio-visual block (*M* = 2.8°, *SD* = 4.5°) to the last 10 trials of the audio-visual block (*M* = −0.2°, *SD* = 1.5°), *t*(17) = 4.27, *p* < 0.001. When feedback was given based on the visual position, the bias significantly increased from the first 10 trials of the audio-visual block (*M* = 7.1°, *SD* = 3.7°) to the last 10 trials of the audio-visual block (*M* = 11.4°, *SD* = 2.9°), *t*(17) = 5.10, *p* < 0.001.

During audio-visual blocks participants received a monetary reward when the error fell below an individual threshold (see section “Audio-Visual Blocks for details). A summary of the received rewards is given in Table 3. A repeated measures MANOVA with factors Feedback Modality (audition or vision) and Visual Reliability (low or high) did neither reveal any significant main effects nor a significant interaction of Feedback Modality and Visual Reliability (see Table 4).

**TABLE 3:** Average reward per session received in audio-visual blocks.

**Reliability**	**FB-modality**	**Absolute mean**	***SD***	**Minimum**	**Maximum**	**Rel. reward**
Visual Rel. low	Audition	6.27	1.86	3.21	10.11	0.58
Visual Rel. high	Audition	6.31	1.92	2.49	10.08	0.58
Visual Rel. low	Vision	6.30	2.34	2.34	10.53	0.58
Visual Rel. high	Vision	6.66	2.39	1.29	10.17	0.62

*The absolute reward is given in €. The last column (Rel. Reward) depicts the reward relative to the maximally possible reward.*

**TABLE 4:** Repeated measures MANOVA on reward in audio-visual blocks.

**Effect**	**Num Df**	**Den Df**	**Pillai test statistic**	**Approximately *F***	***p***
Intercept	1	17	0.94	279.26	<0.001
Feedback modality	1	17	0.02	0.29	0.60
Visual reliability	1	17	0.02	0.37	0.55
Feedback modality: visual reliability	1	17	0.01	0.14	0.72

### Ventriloquism Aftereffect

**FIGURE 5:**
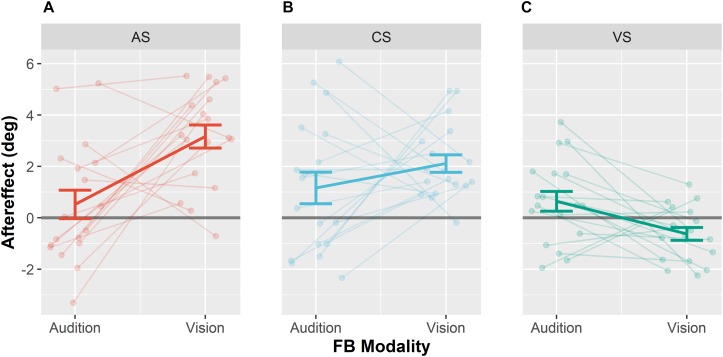
Ventriloquism aftereffects. Aftereffects were collapsed over leftward and rightward audio-visual disparities for the adapted sound AS **(A)**, the control sound CS **(B)**, and the visual stimulus VS **(C)**. Each panel shows aftereffects separately for the conditions Audition FB modality and Vision FB Modality. Individual data are shown with light-colored points and lines whereas sample averages are indicated by dark-colored bold lines. Paired data points (i.e., individual data from a single participant) are connected via lines. Values were calculated as differences between pre- and post-test localization error multiplied with the sign of the audio-visual discrepancy. Thus, shifts in the direction of the competing stimulus during adaptation are positive. Error bars represent the standard error of the mean.

We next examined whether the magnitude of the VAE depended on whether feedback was given based on the visual or based on the auditory position (see Figure 5). In contrast to the standard VAE for the auditory modality (VAE), we will refer to visual aftereffects as *visual Ventriloquism Aftereffect* (vVAE). A reliable VAE was observed for auditory stimuli when vision was the feedback modality. By contrast, no VAE was observed for auditory stimuli when audition was the feedback modality. In none of the two conditions a vVAE significantly different from zero was found. However, mean visual localization responses when vision was the feedback modality compared to when audition was the feedback modality differed significantly. A detailed depiction of mean auditory and visual localization behavior can be found in Supplementary Figures 1, 2. A repeated measures MANOVA (2 × 3 × 6) with factors Feedback Modality (audition or vision), Stimulus Type (AS, CS, and VS) and Stimulus Position (−22.5, −13.5, −4.5, 4.5, 13.5, and 22.5°) revealed a significant interaction of Feedback Modality and Stimulus Type, *F*(2,16) = 7.14, *p* = 0.006. Furthermore, a significant main effect of Stimulus Type was found, *F*(1,17) = 11.07, *p* = 0.001, as well as a significant interaction between Feedback Modality and Stimulus Position, *F*(5,13) = 4.84, *p* = 0.010.

Subsequent pairwise contrasts between the two levels of feedback modality separately calculated for the three levels of Stimulus Type (CS, AS, and VS) revealed that the VAE significantly differed for the AS, *F*(1,17) = 12.7, *p* < 0.001, and the VS, *F*(1,17) = 7.91, *p* = 0.024, such that the VAE for the AS increased when vision was the feedback modality and the vVAE increased when audition was the feedback modality. No effect of feedback modality was found for the CS, *F*(1,17) = 1.36, *p* = 0.259. We additionally performed Bonferroni–Holm corrected *post hoc t*-tests to test whether aftereffects were different from zero for each stimulus type and feedback modality. When vision was the feedback modality, significant aftereffects were found for the AS (*M* = 3.2°, *SD* = 2.4°), *t*(17) = 7.05, *p* < 0.001, and the CS (*M* = 2.1°, *SD* = 1.4°), *t*(17) = 6.21, *p* < 0.001, but not for the VS (*M* = −0.6°, *SD* = 1.1°), *t*(17) = −2.52, *p* = 0.088. No significant aftereffects were found when audition was the feedback modality (see Table 5 for all results).

**TABLE 5:** One-sample *post hoc t* tests comparing VAE and vVAE against zero.

**Stimulus**	**FB-modality**	**Mean**	***SD***	***t***	**Df**	***p***
AS	Audition	0.53	2.36	0.95	17	0.355
AS	Vision	3.17	1.90	7.05	17	<0.001
CS	Audition	1.16	2.62	1.89	17	0.230
CS	Vision	2.11	1.44	6.21	17	<0.001
VS	Audition	0.65	1.63	1.68	17	0.230
VS	Vision	–0.62	1.05	–2.52	17	0.088

*All *p*-values are Bonferroni–Holm corrected. The VAE for the AS and CS as well as the vVAE for the VS were tested against zero depending on whether feedback was based on the position of the auditory or visual stimuli during audio-visual blocks.*

In addition, we performed *post hoc* contrasts (Bonferroni–Holm corrected) separately for each pair of stimuli (CS, AS, and VS) when vision was the feedback modality, to test whether the VAE differed between stimuli. The VAE for the AS was larger than the VAE for the CS, *F*(1,17) = 12.89, *p* = 0.009, and larger than the vVAE for the VS, *F*(1,17) = 46.09, *p* < 0.001. The VAE for the CS was larger than the vVAE for the VS, *F*(1,17) = 32.84, *p* < 0.001.

In order to test whether the influence of the feedback modality was greater for the AS than for the CS, we performed an interaction contrast comparing the difference of the VAE between the conditions vision feedback modality and audition feedback modality for AS (*M* = 2.6°, *SD* = 3.4°) and CS (*M* = 1.0°, *SD* = 3.4°). The difference between VAEs was larger for the AS, *F*(1,17) = 6.65, *p* = 0.020. These results suggest that the effect of feedback modality generalized to the CS only partially.

## Discussion

The present study investigated whether crossmodal recalibration, as operationalized with the VAE, and multisensory integration, as operationalized with the VE, are top–down modulated by feedback. We adapted the standard VAE paradigm by adding feedback during audio-visual adaptation. By giving feedback either based on the position of the auditory stimuli or based on the position of the VS, we were able to assess whether feedback modulates the magnitude of the VE and the VAE. During adaptation, we found that the VE was reduced if feedback was based on the position of the AS. A significant VAE for auditory stimuli was only found when vision was the feedback modality, but not when audition was the feedback modality. Finally, we observed a generalization of the VAE to an untrained sound with a different frequency spectrum.

### Ventriloquism Effect

The analysis of audio-visual trials during adaptation revealed a clear modulation of the VE by feedback. In the ongoing debate of whether the VE is a rather automatic perceptual process (Radeau, 1985; Bertelson and Aschersleben, 1998; Bertelson et al., 2000) or at least to some degree susceptible to top–down processes (Maiworm et al., 2012; Bruns et al., 2014), our results provide further evidence for the latter assumption. The results show similarities to the study of Bruns et al. (2014) in which it was demonstrated that reward can reduce the VE. In their VE paradigm participants received a monetary reward for precise and accurate auditory localization. Any visual bias induced by the VE was, thus, in conflict to the motivational goal of maximizing the reward. Importantly, the amount of reward depended on the hemifield in which the AS was presented. When audio-VS were presented in the hemifield associated with a high reward, the VE was reduced compared to when the audio-VS were presented in the hemifield associated with a low reward. Noteworthy, feedback in our study did not only comprise information about the localization error but also a monetary reward when the localization error fell below a threshold. Thus, our findings extend the results of Bruns et al. (2014) by showing that additional corrective feedback can not only reduce but even extinguish the VE when feedback is based on the AS position. By contrast, feedback and reward increased the VE when they were based on the VS position.

One explanation for the modulation of the VE might be that feedback and reward enhanced auditory processing when audition was the feedback modality. It has been shown that feedback can facilitate visual perceptual learning (Herzog and Fahle, 1997) and that reward can facilitate unisensory discrimination performance (Pleger et al., 2008, 2009). Similarly, feedback in our study might have led to an increase in auditory localization reliability. Given that the size of the VE depends on the relative reliabilities of vision and audition (Ernst and Banks, 2002; Alais and Burr, 2004) this would have resulted in a decreased VE. If this was the case, feedback would have modulated multisensory integration via changed bottom–up processing rather than top–down influences. However, we did not find any differences in unisensory auditory localization reliability (indicated by the variable error) between unimodal trials in the pretest and post-test blocks. Moreover, we did not find differences in localization reliability depending on which modality was feedback-relevant either. In fact, only visual reliability increased from pre- to post-test, regardless of whether audition or vision was feedback-relevant. Thus, changes in reliability-based bottom–up processing should have resulted in an increased VE regardless of which sensory modality was feedback-relevant. Hence, it is unlikely that the decrease or increase of the VE was simply due to altered auditory reliabilities and thus altered bottom–up processing.

Similar to the present findings, recent studies showing a top–down modulation of the VE did not find changes in unisensory processing. Therefore, the authors (Maiworm et al., 2012; Bruns et al., 2014) argued that it might be the process of crossmodal binding itself that is altered by top–down processing. Binding refers here to the problem of inferring whether two signals have a common or distinct source. For both scenarios different strategies are optimal: if the signals emerged from a common cause, a reliability-weighted average is the optimal estimate (Ernst and Banks, 2002; cue integration, see Alais and Burr, 2004). Otherwise, perceptual estimates should be derived separately from unisensory cues (cue segregation). In fact, the brain seems to form estimates for both scenarios at different stages of the cortical hierarchy (Rohe and Noppeney, 2015). In a further processing step, the probability of a common or distinct cause is estimated and a final multisensory percept is formed as a weighted average of the estimates derived by cue segregation and integration (Körding et al., 2007; Beierholm et al., 2010). Each estimate is weighted by the probability of the underlying model (Körding et al., 2007). This approach has proven to describe the VE well in a range of studies (Beierholm et al., 2010; Wozny et al., 2010; Rohe and Noppeney, 2015) and is referred to as “causal inference” (Körding et al., 2007).

In fact, decreasing the binding tendency and relying on unisensory estimates would have been a beneficial strategy in our paradigm. The shift in localization behavior during bimodal trials toward the feedback-relevant sensory modality indicates that participants picked up the relation between sensory modality and feedback. Thus, the feedback-relevant modality might have been identified as task-relevant. It is known that task relevance modulates auditory and visual weights in multisensory integration independently from bottom–up factors such as reliability (Rohe and Noppeney, 2016). This up- or down-weighing might be mediated by attentional shifts toward one modality (Mozolic et al., 2007; Padmala and Pessoa, 2011) or reallocation of cognitive control resources (Pessoa, 2009) to the feedback-relevant modality.

Although the VE seems to be independent from spatial attention, several examples exist in multisensory integration where attentional shifts to a specific modality (rather than to a specific location) lead to decreased integration of task-irrelevant stimuli presented in another modality (Johnson and Zatorre, 2005; see Keil and Senkowski, 2018 for a review). Recent studies have demonstrated that audio-visual integration occurs at different stages of the cortical hierarchy in parallel (Calvert and Thesen, 2004; Rohe and Noppeney, 2015) and that these different stages are associated with distinct computational principles (Rohe and Noppeney, 2015, 2016). It has been argued that multisensory integration associated with late processing stages might be prone to top–down modulation whereas integration associated with early stages might be more or less automatic (Koelewijn et al., 2010). Following this argument, feedback might have modulated late stages of the cortical hierarchy which are linked to audio-visual percepts based on causal inference (Rohe and Noppeney, 2015; Aller and Noppeney, 2019).

The importance of top–down processing seems to increase when tasks include motivational incentives, monetary reward (Rosenthal et al., 2009; Bruns et al., 2014), emotional valence (Maiworm et al., 2012) or avoiding harm (Shapiro et al., 1984). For instance, the sound-induced flash illusion was only susceptible to feedback when feedback was accompanied by a reward (Rosenthal et al., 2009). Similarly, explicit knowledge of a spatial discrepancy between audition and vision did not alter the VE (Bertelson and Aschersleben, 1998). However, here we show that corrective feedback paired with a monetary reward clearly increased or decreased the VE depending on whether audition or vision was feedback-relevant.

### Ventriloquism Aftereffect

In order to maintain accuracy, the perceptual system must infer which sensory modality is inaccurate and to what extent. Ideally, each sensory modality should be recalibrated according to the magnitude of its inaccuracy. In the standard VAE paradigm audition is calibrated toward vision which can provide internal consistency (Radeau and Bertelson, 1974; Kopco et al., 2009; Zaidel et al., 2011; Pages and Groh, 2013). However, when audition is accurate, and vision is biased, recalibrating audition toward vision introduces inaccuracies in the perceptual system.

As predicted by the assumption that the maintenance of accurate sensory modalities is the primary objective of crossmodal recalibration (Di Luca et al., 2009; Block and Bastian, 2011; Zaidel et al., 2013), we found that feedback based on audition can suppress the VAE. Hence, the perceptual system did not recalibrate auditory spatial perception when feedback implied that audition was already accurate. By contrast, when vision was feedback-relevant a substantial VAE of 23.5% of the size of the audio-visual discrepancy (13.5°) was found. We did not provide direct sensory feedback (as often used in sensory-motor adaptation paradigms) about the true stimulus position which would have allowed the perceptual system to infer sensory prediction errors in a bottom–up manner (Izawa and Shadmehr, 2011). Instead, a centrally presented arrow indicated magnitude and direction of the localization error, requiring participants to consciously infer the semantic meaning of the feedback. Hence, feedback must have modulated crossmodal recalibration in a top–down manner.

In contrast to our assumption that external accuracy drives recalibration, one could argue that the VAE in our study followed the principles of reliability-based adaptation (Ghahramani et al., 1997; van Beers et al., 2002; Burge et al., 2010; Makin et al., 2013). Feedback might have facilitated unisensory auditory processing, as has been shown in unimodal experiments (Pleger et al., 2008, 2009), and, thereby, increased auditory reliability. Thus, according to this assumption audition would be weighted more in the recalibration process, leading to less recalibration. Analogously to our results for the VE, it is unlikely that changes in reliability could explain the results as we did not find an increase in auditory localization reliability between pretest and post-test and reliability in AS trials did not differ depending on which sensory modality was feedback-relevant.

Zaidel et al. (2013) proposed that external feedback invokes a second recalibration process which is superimposed on unsupervised crossmodal recalibration without external feedback and relies on cue reliabilities. Hence, both processes occur in parallel when feedback is present. According to Zaidel et al. (2013), feedback based on the less reliable sensory modality leads to increased supervised recalibration to an extent that outreaches the effect of unsupervised recalibration. Importantly, supervised and unsupervised recalibration result in shifts in opposite directions for the cue that feedback is based on. This results in an overall recalibration of the less reliable sensory modality away from the reliable sensory modality (negative aftereffect). In contrast to Zaidel et al. (2013), we did not find any significant negative aftereffects although audition was clearly less reliable than vision (Figure 3).

Interestingly, Pages and Groh (2013) argued that the VAE without external feedback might be a form of supervised learning itself, whereby vision functions as the supervisor for audition. In line with this assumption, they demonstrated that a VAE only occurred when the VS were presented long enough for participants to perform saccades toward them. When VS were extinguished before participants could accomplish saccades, no VAE occurred. Our results support the assumption that external feedback in audio-visual spatial recalibration needs to provide information about the magnitude and direction of the localization error in order to be effective.

We did not observe a recalibration of vision (a vVAE) in our study, neither when audition was feedback-relevant nor when vision was feedback-relevant. There are only a few reports of vVAEs (Radeau and Bertelson, 1976; Lewald, 2002), and even prism adaptation for several weeks usually does not result in visual aftereffects (Welch, 1978). Hence it is questionable whether it is possible to induce visual aftereffects through audio-visual adaptation at all (Welch, 1978; Lewald, 2002; Zaidel et al., 2011). Ernst and Di Luca (2011) have argued that in order to stay accurate, the perceptual system has to infer to which extent a sensory discrepancy can be attributed to individual inaccuracies of the contributing sensory modalities. As there is no direct information in the sensory cues allowing to assess accuracy, a way to resolve this assignment problem is to form prior beliefs about the probability of a sensory cue to be biased (bias prior). Sensory recalibration then only depends on the ratio of the bias priors. The lack of visual aftereffects could be explained by a remarkably small bias prior for vision. Our results indicate that it might not be possible to update this bias prior on the time scale and by the type of external feedback that was used in the present study (fixed prior, Van Wassenhove, 2013). It has been argued that vision, as the most reliable spatial sense, serves as a reference to calibrate the other senses (Radeau and Bertelson, 1974; Knudsen and Knudsen, 1989; Bertelson et al., 2006; Kopco et al., 2009). If the visual system serves as a reference for other sensory modalities, a fixed prior is beneficial to avoid unstable visual sensory estimates in an ever-changing multisensory environment.

To efficiently recalibrate, the perceptual system must infer whether the discrepancy between two sensory cues is due to sensory inaccuracies or whether the cues simply reflect distinct sources. Ideally, recalibration should only occur when a discrepancy can be attributed to sensory inaccuracies (Mahani et al., 2017). We argue that during bimodal trials the VE might have decreased when feedback was based on audition relative to when feedback was based on vision due to a decreased binding tendency which manifests in a reduced prior probability of a common cause (Körding et al., 2007). Hence the increased probability of distinct causes in bimodal trials might have also reduced recalibration. A recent fMRI study (Zierul et al., 2017) showed that the VAE is associated with activity changes in the planum temporale, a region which has also been associated with the VE (Bonath et al., 2007), suggesting that neural circuitries involved in the VE and VAE are overlapping (see also Park and Kayser, 2019). Thus, causal inference processes might affect the VAE via the same neural circuitry as the VE (Rohe and Noppeney, 2015).

In contrast to previous studies (Recanzone, 1998; Lewald, 2002; Bruns and Röder, 2015) we found a significant transfer of the VAE to an untrained AS (see Figure 5). However, there is an ongoing debate whether the VAE is sound frequency-specific (Recanzone, 1998; Lewald, 2002; Bruns and Röder, 2015) or generalizes across sound frequencies (Frissen et al., 2003, 2005), and generalization might depend on the sensory context in which audio-visual adaptation takes place (Bruns and Röder, 2019b). Although a significant VAE emerged for the CS, our results indicate that feedback had a specific effect on the AS used during adaptation (AS) as the difference of the VAE between the conditions vision feedback modality and audition feedback modality was significantly reduced for the auditory CS which was only presented during pre- and post-test.

In summary, the suppression of the VAE by feedback based on audition challenges the assumption that the VAE is an automatic process which is independent from top–down influences (Epstein, 1975; Radeau and Bertelson, 1978; Passamonti et al., 2009). Although the VAE readily occurs when top–down processing can be excluded (Passamonti et al., 2009), our findings demonstrate that the perceptual system can flexibly integrate external feedback into the process of crossmodal recalibration, highlighting the importance of external accuracy as a driving factor for crossmodal recalibration.

## Data Availability Statement

The datasets generated for this study are available on request from the corresponding author.

## Ethics Statement

The studies involving human participants were reviewed and approved by the local ethics commission of the Faculty of Psychology and Human Movement of the University of Hamburg and were conducted in accordance with the Declaration of Helsinki. The participants provided their written informed consent to participate in this study.

## Author Contributions

All authors conceptualized the study and revised and approved the final manuscript. AK collected and analyzed the data. AK and PB wrote the manuscript.

## Conflict of Interest

The authors declare that the research was conducted in the absence of any commercial or financial relationships that could be construed as a potential conflict of interest.
