# Supplementary material for: Feedback Modulates Audio-Visual Spatial Recalibration
Source: Front Integr Neurosci. 2020 Jan 17;13:74. doi: 10.3389/fnint.2019.00074 (PMC6979315; doi:10.3389/fnint.2019.00074)
Supplement: Supplementary file 1 [file Image_1.pdf]

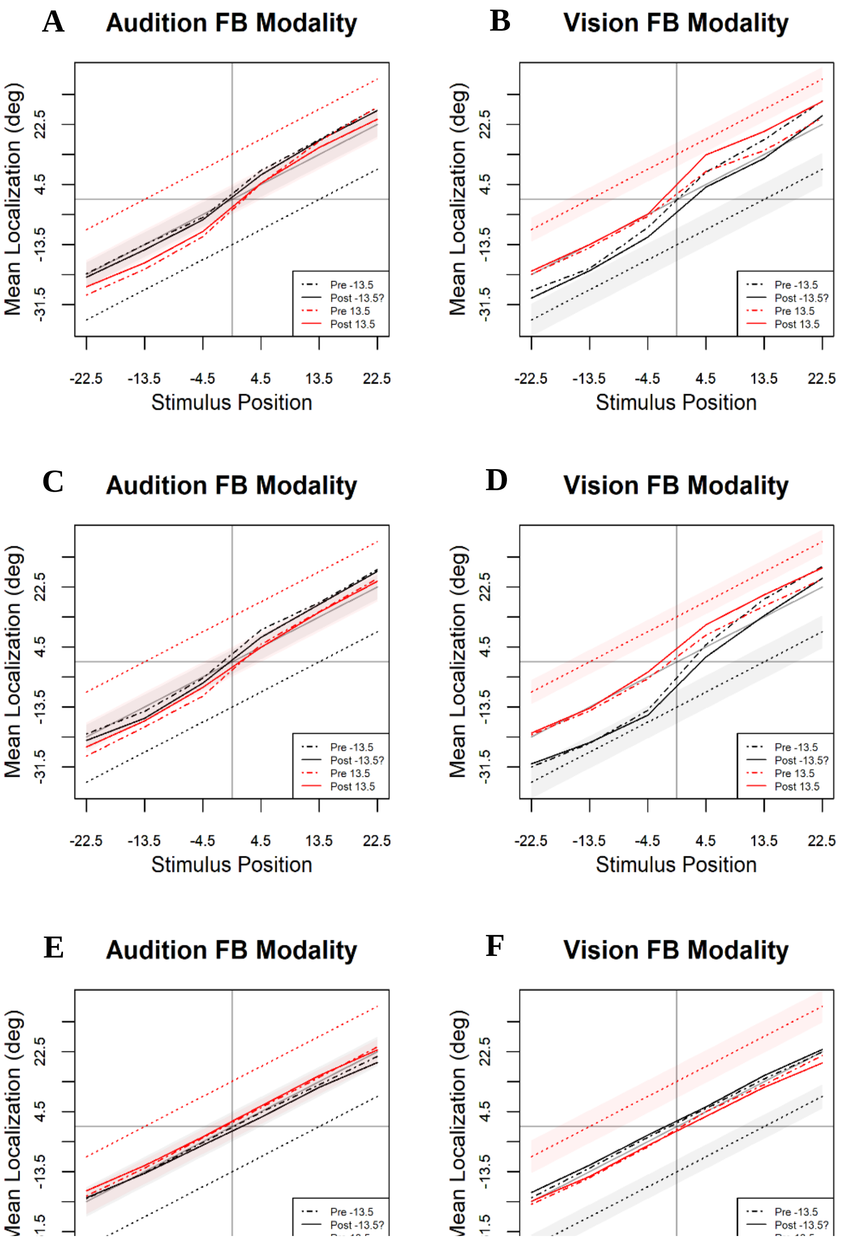

Supplementary Figure 1. Auditory and visual localization behavior for the adapted sound (A and B), the control sound (C and D) and the visual stimulus (E and F) in unimodal blocks. The first column shows results when audition was the feedback modality, and the second column shows the results when vision was the feedback modality. Each panel shows results separately for pretest (dashed lines) and posttest (solid lines). Red lines represent sessions where the audio-visual disparity during adaptation was to the right, and black lines show results for sessions where the audio-visual disparity was to the left. Shaded areas represent reward zones for the respective conditions.
