# Supplementary material for: Feedback Modulates Audio-Visual Spatial Recalibration
Source: Front Integr Neurosci. 2020 Jan 17;13:74. doi: 10.3389/fnint.2019.00074 (PMC6979315; doi:10.3389/fnint.2019.00074)
Supplement: Supplementary file 2 [file Image_2.pdf]

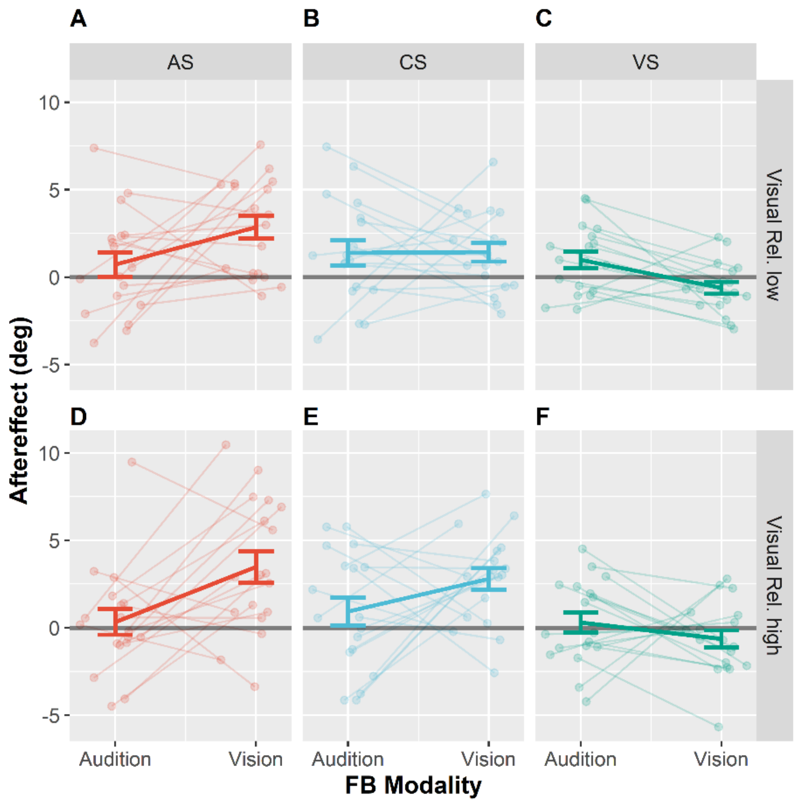

Supplementary Figure 2. Ventriloquism aftereffects for different levels of visual reliability. Aftereffects are shown in separate panels for the different stimulus types (AS in panel A and D, CS in panel B and E, VS in panel C and F) and collapsed over leftward and rightward audio-visual disparities. Mean ventriloquism aftereffects for different levels of visual reliability are depicted in separate rows (Visual Rel. low in panel A-C and Visual Rel. high in panel D-E). Each panel shows aftereffects separately for the conditions Audition FB modality and Vision FB Modality. Individual data is shown with light-colored points and lines whereas sample averages are indicated by dark-colored bold lines. Paired data points (i.e., individual data from a single participant) are connected via lines. Values were calculated as differences between pre- and posttest localization errors multiplied with the sign of the audio-visual discrepancy. Thus, shifts in the direction of the competing stimulus during adaptation are positive. Error bars represent the standard error of the mean.
