# Supplementary material for: Feedback Modulates Audio-Visual Spatial Recalibration
Source: Front Integr Neurosci. 2020 Jan 17;13:74. doi: 10.3389/fnint.2019.00074 (PMC6979315; doi:10.3389/fnint.2019.00074)
Supplement: Supplementary file 3 [file Table_1.pdf]

Supplementary Table 1. Total number of participants for each combination of sound frequency of the adapted sound, FB modality and visual reliability.

		Audition FB Modality		Vision FB Modality	
		Visual Rel. low	Visual Rel. high	Visual Rel. low	Visual Rel. high
Sound Frequency Adapted Sound	250 Hz	5	5	5	3
	500 Hz	4	4	6	4
	1000 Hz	5	5	3	5
	2000 Hz	4	4	4	6
