# Supplementary material for: Feedback Modulates Audio-Visual Spatial Recalibration
Source: Front Integr Neurosci. 2020 Jan 17;13:74. doi: 10.3389/fnint.2019.00074 (PMC6979315; doi:10.3389/fnint.2019.00074)
Supplement: Supplementary file 4 [file Table_2.pdf]

Supplementary Table 2. **Total number of participants for each combination of session number, FB modality and visual reliability.** The session numbers reflect the temporal order of sessions for each participant.

		Audition FB Modality		Vision FB Modality	
		Visual Rel. low	Visual Rel. high	Visual Rel. low	Visual Rel. high
Session Number	1	5	4	4	5
	2	3	5	5	5
	3	5	5	4	4
	4	5	4	5	4
