# Supplementary material for: Feedback Modulates Audio-Visual Spatial Recalibration
Source: Front Integr Neurosci. 2020 Jan 17;13:74. doi: 10.3389/fnint.2019.00074 (PMC6979315; doi:10.3389/fnint.2019.00074)
Supplement: Supplementary file 5 [file Table_3.pdf]

Supplementary Table 3. Total number of participants for each combination of audio-visual discrepancy, FB modality and visual reliability.

		Audition FB Modality		Vision FB Modality	
		Visual Rel. low	Visual Rel. high	Visual Rel. low	Visual Rel. high
Audio-visual Discrepancy	13.5°	5	5	5	3
	-13.5°	4	4	6	4
